# Differential binding of human and murine IgGs to catalytic and cell wall binding domains of *Staphylococcus aureus* peptidoglycan hydrolases

**DOI:** 10.1038/s41598-021-93359-6

**Published:** 2021-07-05

**Authors:** Min Wang, Sanne van den Berg, Yaremit Mora Hernández, Aafke Hinke Visser, Elias Vera Murguia, Dennis G.A.M. Koedijk, Channah Bellink, Hilde Bruggen, Irma A. J. M. Bakker-Woudenberg, Jan Maarten van Dijl, Girbe Buist

**Affiliations:** 1 Department of Medical Microbiology, University of Groningen, University Medical Center Groningen, Hanzeplein 1, HPC EB80, P.O. box 30001, 9700 RB Groningen, the Netherlands; 2 Department of Medical Microbiology and Infectious Diseases, Erasmus University Medical Center, Rotterdam, The Netherlands

**Keywords:** Microbiology, Bacteria, Bacteriology, Pathogens

## Abstract

*Staphylococcus aureus* is an opportunistic pathogen causing high morbidity and mortality. Since multi-drug resistant *S. aureus* lineages are nowadays omnipresent, alternative tools for preventive or therapeutic interventions, like immunotherapy, are urgently needed. However, there are currently no vaccines against *S. aureus*. Surface-exposed and secreted proteins are regarded as potential targets for immunization against *S. aureus* infections. Yet, many potential staphylococcal antigens of this category do not elicit protective immune responses. To obtain a better understanding of this problem, we compared the binding of serum IgGs from healthy human volunteers, highly *S. aureus*-colonized patients with the genetic blistering disease epidermolysis bullosa (EB), or immunized mice to the purified *S. aureus* peptidoglycan hydrolases Sle1, Aly and LytM and their different domains. The results show that the most abundant serum IgGs from humans and immunized mice target the cell wall-binding domain of Sle1, and the catalytic domains of Aly and LytM. Interestingly, in a murine infection model, these particular IgGs were not protective against *S. aureus* bacteremia. In contrast, relatively less abundant IgGs against the catalytic domain of Sle1 and the N-terminal domains of Aly and LytM were almost exclusively detected in sera from EB patients and healthy volunteers. These latter IgGs may contribute to the protection against staphylococcal infections, as previous studies suggest that serum IgGs protect EB patients against severe *S. aureus* infection. Together, these observations focus attention on the use of particular protein domains for vaccination to direct potentially protective immune responses towards the most promising epitopes within staphylococcal antigens.

## Introduction

*Staphylococcus aureus* is a leading pathogen in human beings and livestock. In humans, *S. aureus* causes a wide range of diseases that vary from mild skin and soft tissue infections to life-threatening diseases, such as pneumonia, bacteremia and infective endocarditis. Importantly, *S. aureus* is carried asymptomatically by approximately 20–30% of the healthy human population^1–4^*.* Previous studies have shown that nasal carriage of *S. aureus* increases the risk of infection by this pathogen, but these studies also indicated that the course of such infections is usually less severe in carriers^5,6^. This indicates that carriage of *S. aureus* may elicit some adaptive immunity against this pathogen. Furthermore, it was observed that patients with the genetic blistering disease epidermolysis bullosa (EB), whose chronic wounds are heavily colonized by *S. aureus*, display elevated levels of potentially protective anti-staphylococcal serum immunoglobulin G (IgG) compared to healthy volunteers^7,8^. In particular, since these EB patients rarely suffer from severe invasive staphylococcal infections^9^, it was previously proposed that their high exposure to multiple types of *S. aureus* over extended periods of time elicited protective humoral immune responses, as reflected by elevated levels of IgG_1_ and IgG_4_ class antibodies against *S. aureus*^7,10,11^. On the other hand, *S. aureus* has evolved multiple highly effective mechanisms to evade the human immune defenses^12^. For many years, the resulting infections could be effectively treated with antibiotics. However, this is becoming increasingly difficult due to the spread of antibiotic resistant *S. aureus* lineages, both in hospitals and the community, as exemplified by methicillin-resistant *S. aureus* (MRSA)^13,14^. Therefore, there is a need to develop effective alternative treatments to fight staphylococcal infections, which focuses interest on novel immunotherapeutic approaches.

Active immunization is considered as a very effective approach to prevent infectious diseases^15^. For instance, highly effective vaccines have been used over many years to protect humans against infections by *Streptococcus pneumoniae*^16^. However, the development of protective vaccines against *S. aureus* has turned out to be more challenging as none of the candidate vaccines has, thus far, successfully passed phase III clinical trials^17^. These candidate vaccines included various different *S. aureus* antigens, such as capsular polysaccharides (types 5 and 8), wall teichoic acids and proteinaceous virulence factors. Amongst the latter were proteins like the clumping factor A, fibronectin-binding proteins, and the iron-regulated surface determinant B. These proteins were included in the vaccine formulations as single or combined antigens^18–21^. However, in spite of the protective effects observed in murine models, the tested candidate vaccines failed to protect humans against *S. aureus* infection^22^. Important underlying reasons for the inefficacy of candidate anti-staphylococcal vaccines could be the high heterogeneity of antigens expressed by different *S. aureus* lineages, as well as host-specific responses to the applied antigens. In this respect, it should be noted that the heterogeneity of presented antigens is actually much higher than generally perceived, since individual proteins can present different immunogenic epitopes. While some of these epitopes may elicit protective antibody responses, the recognition of other epitopes may not lead to a response that protects against staphylococcal infections, as was previously demonstrated for the immunodominant staphylococcal antigen A (IsaA)^23,24^. It thus seems that the high heterogeneity in possible epitopes presented to the human immune system represents a significant challenge for development of an effective vaccine against *S. aureus*^25^.

*S. aureus* produces a large number of different cell surface-bound and secreted proteins, which play important roles in the acquisition of nutrients, host colonization or invasion^26,27^. In general, surface-exposed proteins are regarded as preferred potential targets for active, as well as passive immunization against *S. aureus.* This relates to the fact that cell wall-bound proteins may present the bacteria directly to the host immune system^26,28^. Most peptidoglycan hydrolases are cell surface proteins that bind covalently or non-covalently to the peptidoglycan. These proteins play vital roles in the growth, division and separation of bacterial cells, as well as the general cell wall turnover^29^. It has previously been reported that the amidase domain of the major *S. aureus* peptidoglycan hydrolase Atl can elicit protective immunity against this pathogen in a murine infection model^30^, either through the staphylococcal opsonization by anti-Atl antibodies and subsequent immune clearance or through inactivation of Atl by such antibodies. This implies that peptidoglycan hydrolases could be potential targets for vaccine development. In fact, our previous investigations on the cell surface proteome of *S. aureus* showed that the expression of peptidoglycan hydrolases is highly conserved in *S. aureus*, including clinical strains. Remarkably, five of the seven commonly identified extra-cytoplasmic proteins of *S. aureus* are peptidoglycan hydrolases, namely Aly, IsaA, LytM, SsaA-B and the SsaA-like protein, as was shown in in vitro studies^25^. Further, it has been reported that some of the staphylococcal peptidoglycan hydrolases, like Sle1^31^, SsaA2^31^ and LytM^32^ are highly immunogenic. It thus appears that peptidoglycan hydrolases are possible candidate proteins for inclusion in an anti-staphylococcal vaccine.

Since relatively little is known about the possibilities to use peptidoglycan hydrolases for vaccination against *S. aureus*, the present study was aimed at investigating the immunogenicity of three representative enzymes, namely Sle1 (also referred to as Aaa or SA0423), Aly (SA2437) and LytM (SA0265). In particular, we investigated whether immunization with these three proteins would be protective against *S. aureus* in a murine infection model. Afterwards, we evaluated possible differences in the recognition of distinct domains of Sle1, Aly and LytM by human and murine IgGs. Briefly, the results show that, in particular, the cell wall-binding domain of Sle1, and the catalytic domains of Aly and LytM represent the most immunogenic regions of the investigated proteins. However, the extent to which they elicit IgG responses depends on the individual human or murine host.

## Results

### Modular composition of the peptidoglycan hydrolases Sle1, Aly and LytM

**Figure 1:**
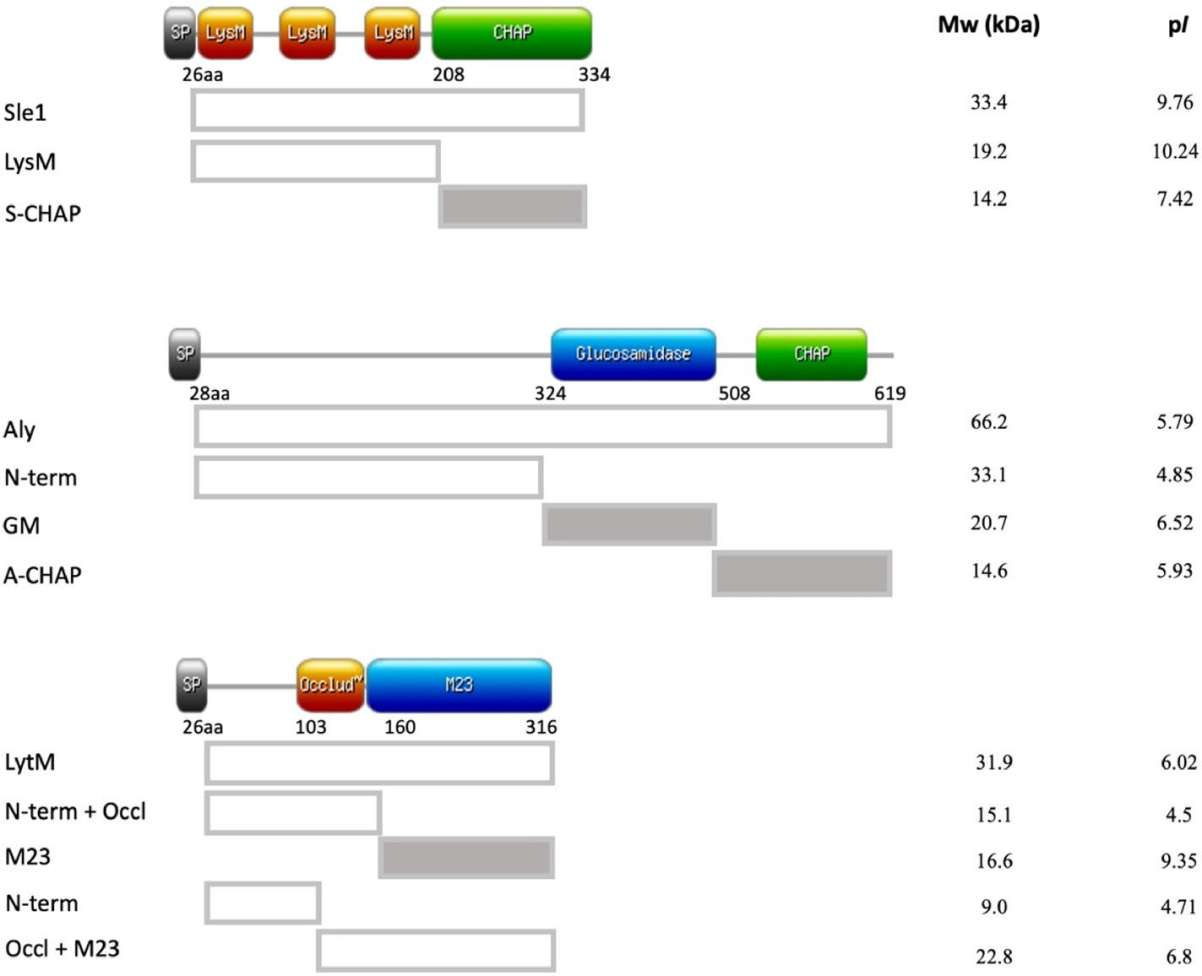
Schematic representation of the Sle1, Aly, LytM domains and their specific characteristics. The diagrams on the left of the image indicate the specific domains of Sle1, Aly and LytM. The position of the first amino acid residue of each domain, as well as the position of the C-terminal residue of each protein are indicated. In addition, the isoelectric points (*pI*) and molecular weights (Mw in kDa) of the full-size proteins and the distinguished domains are indicated. The catalytic domains (CHAP, GM and M23) are indicated in grey shading.

To investigate the immunogenicity of specific domains of the peptidoglycan hydrolases Sle1, Aly and LytM, we first performed a bioinformatics analysis that distinguished different structural domains in these proteins. For this purpose, we performed blast searches, consulted the Pfam and Uniprot databases and used information from previously published studies^33–35^. This resulted in the distinction of specific cell wall binding and peptidoglycan hydrolase domains as schematically represented in Fig. 1. In particular, two domains were distinguished for Sle1, namely a cell wall-binding domain containing three ‘Lysin Motif’ (LysM) repeats, and a so-called ‘cysteine, histidine-dependent amidohydrolases/peptidases’ (CHAP) domain that contains the active site. The CHAP domain of Sle1 (here referred to as S-CHAP) is known to cleave the lactyl bonds between N-acetylmuraminic acid and L-alanine^33,34^. For the hypothetical peptidoglycan hydrolase Aly, we distinguished three different domains. These include an N-terminal domain with unknown function, followed by a glucosaminidase (GM) domain and a C-terminal CHAP domain (here referred to as A-CHAP; Fig. 1). Although the CHAP domains of Sle1 and Aly are homologous, sharing 38% of amino acid sequence identity (Supplemental Fig. 1), they have different isoelectric points of 7.42 and 5.93, respectively. Lastly, three domains were distinguished in LytM. This peptidoglycan hydrolase contains an N-terminal domain with as yet unknown function, followed by a so-called ‘occluding domain’ and a C-terminal ‘Peptidase M23’ (M23) domain. The M23 domain can hydrolyze the pentaglycine-interpeptide bridge of peptidoglycan^35^. Blast analysis showed that the M23 domain of LytM shares 41% amino acid sequence identity to the corresponding domain of the LytU protein (Supplemental Fig. 1B). Of note, a previous study has shown that the occluding domain may block the peptidoglycan hydrolyzing activity of the M23 domain^35^.

### Cell wall hydrolase activity of Sle1, Aly, LytM and their domains

**Figure 2:**
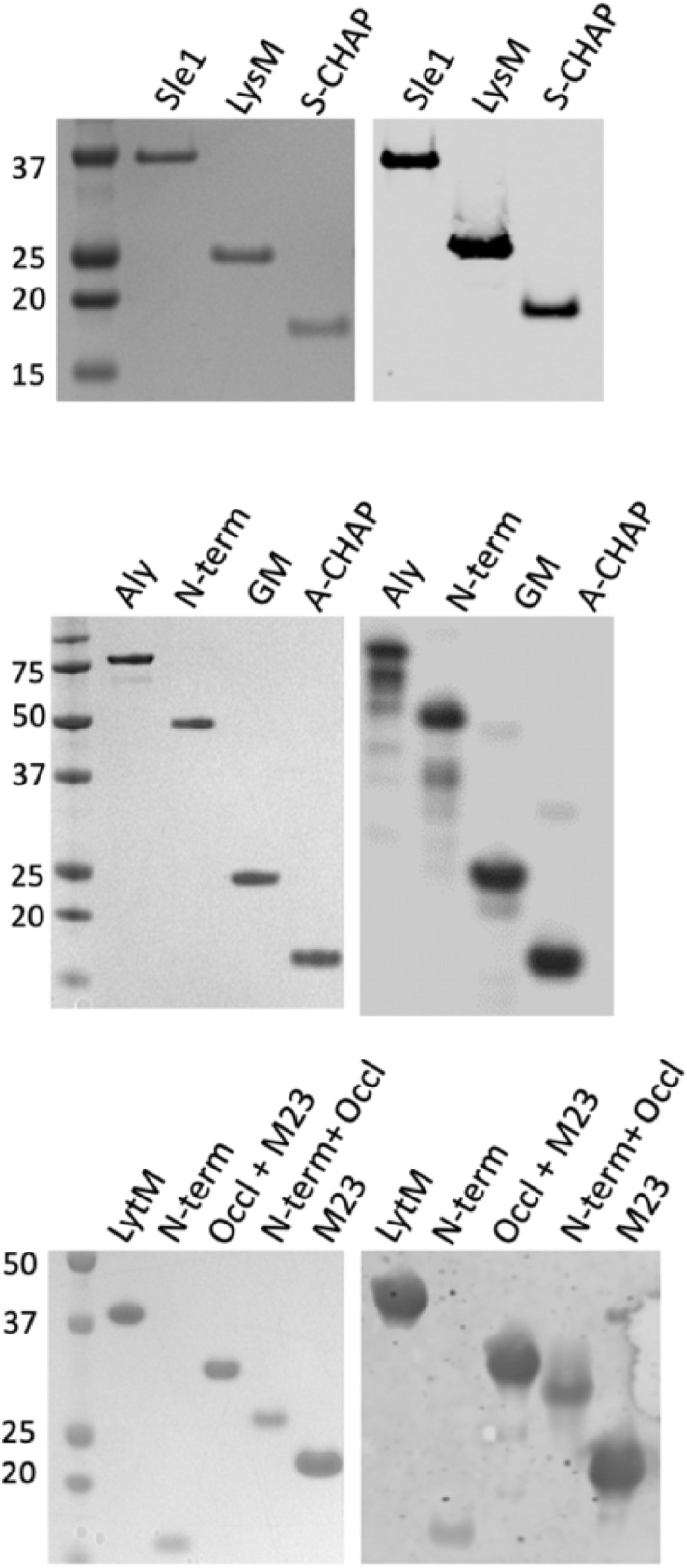
Purification of Sle1, Aly and LytM and their domains. The Sle1, Aly and LytM proteins and their domains were expressed in *L. lactis* PA1001 with a C-terminal His_6_-tag and purified from the growth medium fraction by metal-affinity chromatography. The purified proteins and domains were further analyzed by LDS-PAGE and SimplyBlue staining (left panels), or Western blotting and subsequent immunodetection with anti-His_6_ antibodies (right panels). The positions of molecular weight markers are indicated in kDa. For original gels and Western blots see Supplemental Figs. 2A and B, respectively. The LDS-PAGE and Western blotting experiments were replicated twice.

To study the possible cell wall hydrolase activity of Sle1, Aly, LytM and their domains, as well as their immunogenicity, the respective proteins and domains were expressed in *L. lactis.* To this end, these proteins and domains were N-terminally fused to an appropriate signal peptide and C-terminally to a His_6_-tag, allowing their purification from the culture media or urea-treated cells by metal-affinity chromatography. Subsequent LDS-PAGE analysis showed that the respective proteins and domains were pure upon isolation. Most of the isolated proteins and domains showed the expected mobility upon LDS-PAGE, in accordance with their molecular weight (Fig. 2, left panels). However, the combined N-terminal *plus* occluding domains of LytM, and the N-terminal domain of Aly showed a lower mobility on LDS-PAGE than expected based on the calculated molecular weight (Fig. 2, left panels). This may be due to their low isoelectric points. Upon Western blotting and immunodetection of the proteins and their domains with anti-His_6_-specific antibodies, some minor degradation products were observed for the purified Aly protein and its specific domains (Fig. 2, right panels).

**Figure 3:**
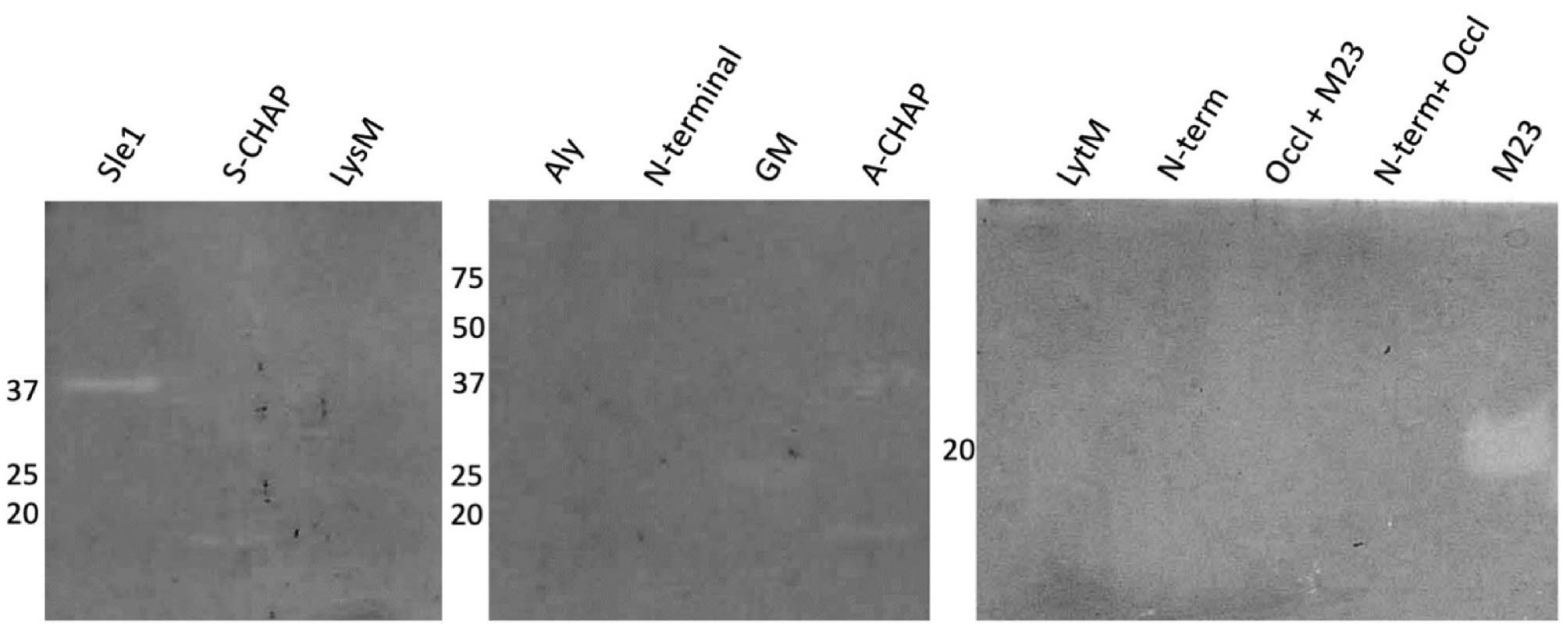
Zymographic detection of cell wall hydrolytic activity of purified proteins or domains. Cell wall hydrolytic activity of the purified Sle1, Aly or LytM proteins and their domains was investigated by zymography. To this end, SDS-PAGE was performed with gels containing 0.1% (w/v) autoclaved cell wall fragments of *S. aureus* RN4220. Upon electrophoresis, the gels were incubated in renaturation buffer for 16 to 48 h at 37 °C, and subsequently stained with methylene blue. The molecular weights (kDa) of the purified proteins and domains are indicated on the left. For original gels see Supplemental Fig. 2C. The zymography experiments were replicated twice.

To analyze the cell wall hydrolase activities of the purified Sle1, Aly and LytM proteins, as well as their domains, zymography experiments were performed. To this end, autoclaved *S. aureus* RN4220 cells or *M. lysodeikticus* cells were incorporated in gels for SDS-PAGE, and the purified proteins and domains were subsequently separated using these gels. Upon in-gel renaturation of the separated proteins and domains and incubation at 37 °C, peptidoglycan degradation was visualized by gel staining with methylene blue. Thus, it was observed that the full-size Sle1 protein and its S-CHAP domain specifically hydrolyzed *S. aureus* cell walls (Fig. 3), but not the *M. lysodeikticus* cell walls (not shown). In contrast, the full-size Aly and LytM proteins did not show any activity towards either of the two potential substrates. Nevertheless, the GM and A-CHAP domains of Aly, and the C-terminal M23 domain of LytM did hydrolyze *S. aureus* cell walls (Fig. 3), but not *M. lysodeikticus* cell walls (not shown). These results show that all three enzymes are capable of peptidoglycan hydrolysis, either as a full-size mature protein, or as a catalytic domain separated from other protein domains. While this activity was previously demonstrated for Sle1 and LytM, the cell wall hydrolase activity of Aly had not yet been experimentally demonstrated.

### Immunization with purified Sle1, Aly or LytM antigens does not protect against *S. aureus* bacteremia in a murine infection model

**Figure 4:**
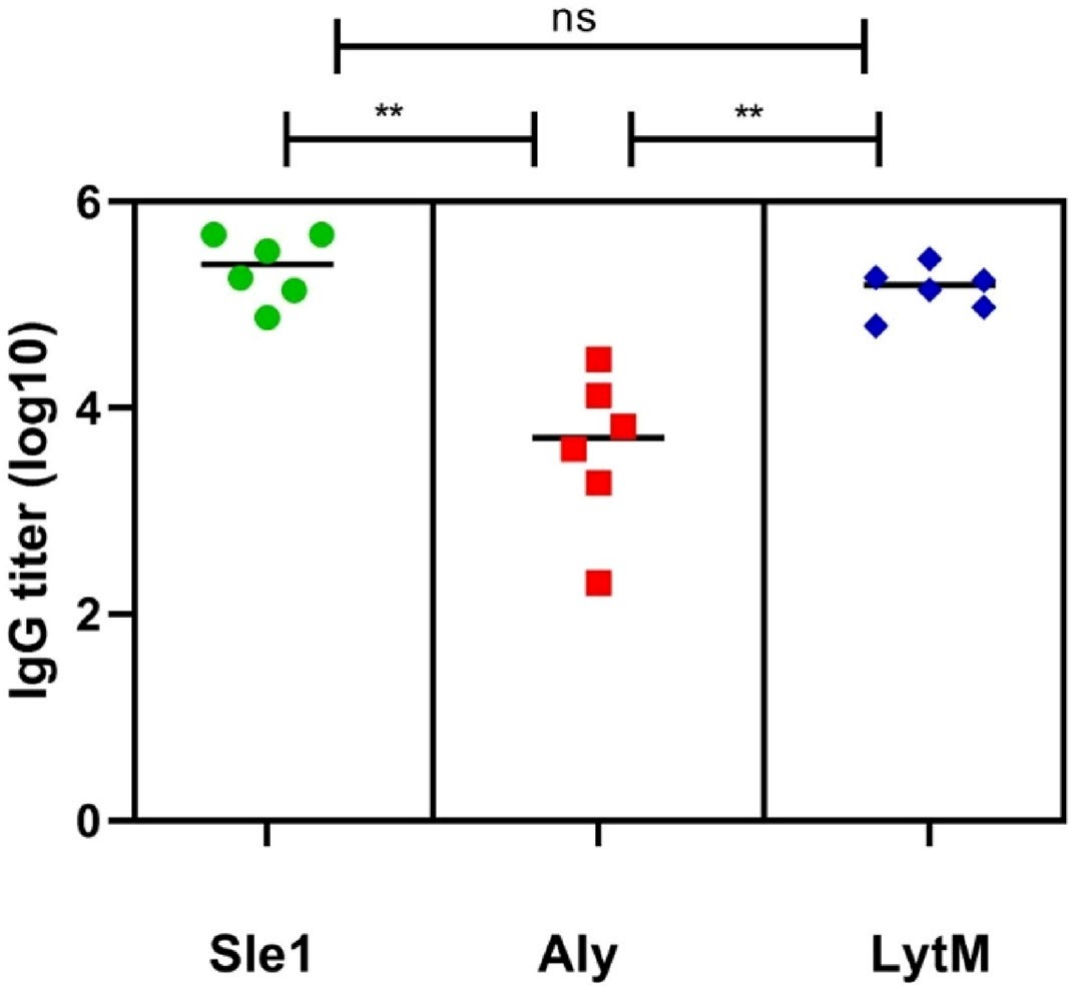
Sle1-, Aly- and LytM-specific IgG titers in sera of immunized mice. Mice were immunized with the full-size Sle1, Aly or LytM proteins (25 µg) at days − 28, − 21 and − 14 prior a challenge with *S. aureus* isolate P (see Fig. 5). For placebo immunizations, mice received an injection with phosphate-buffered saline. On day -1, the Sle1-, Aly- and LytM-specific IgG titers were determined by ELISA. Horizontal lines represent median values and each symbol represents a separate sample. Green symbols, Sle1-immunized mice; red symbols, Aly-immunized mice; blue symbols, LytM-immunized mice. The determined IgG titers for each protein were corrected for the background IgG binding in sera from the placebo-immunized mice. The statistical significance of differences in the IgG titers of sera from the three groups of immunized mice was verified by one-way ANOVA (P < 0.001), and by using the Bonferroni correction to determine the significance of differences between the three groups (**, P < 0.0002).

**Figure 5:**
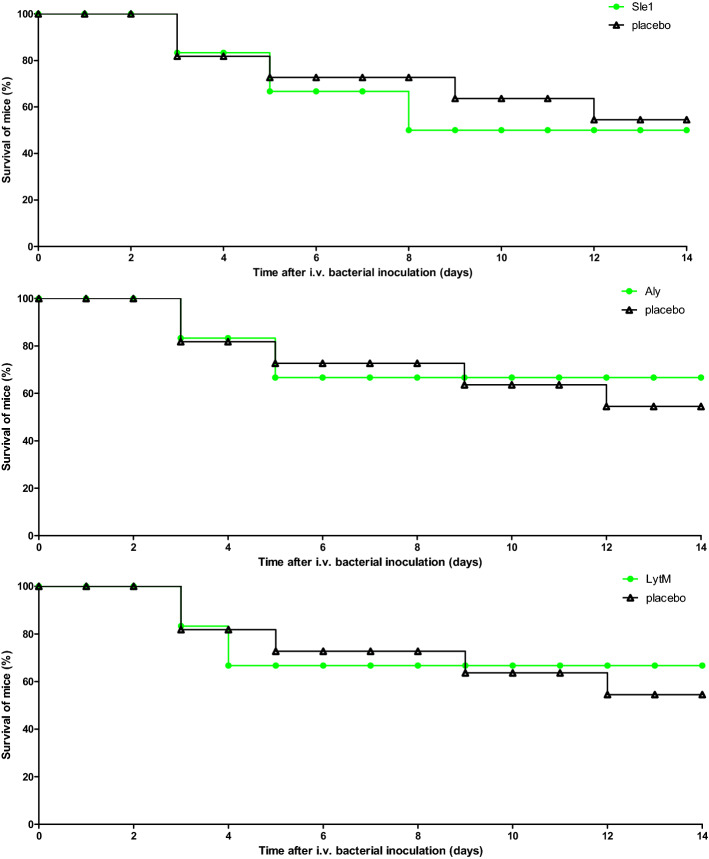
Survival rates of immunized mice challenged with *S. aureus*. Mice immunized with Sle1, Aly or LytM (n = 6 immunized mice per antigen, n = 11 placebo-immunized mice) were subjected to intravenous inoculation of *S. aureus* isolate P (3 × 10^5 ^ CFU). Subsequently, the mice were observed for 14 days. Green symbols, Sle1-, Aly- or LytM-immunized mice; black symbols, placebo group. A significant difference was not observed in animal survival rate (P > 0.5, log rank test).

To investigate whether vaccination with Sle1, Aly or LytM may elicit protective immune responses against *S. aureus* infection, the three purified proteins were separately used to immunize pathogen-free female BALB/cBYJ mice. Blood samples were collected after immunization, and the specific serum IgG levels were determined by ELISA. This showed that each of these antigens elicited humoral immune responses (Fig. 4). Interestingly, the highest specific IgG levels were detected for Sle1. The LytM- and Aly-specific IgG levels were, respectively, around 2- and 60-fold lower than the Sle1-specific IgG levels (Fig. 4). Despite the significant Sle1-, Aly- or LytM-specific IgG levels that were detectable upon immunization of mice with these proteins, a subsequent challenge experiment with the methicillin-sensitive *S*. *aureus* isolate P showed that this immune response did not protect the mice against dying from staphylococcal bacteremia (Fig. 5).

### Distinction of Sle1, Aly and LytM epitopes recognized by human sera and sera from immunized mice

**Figure 6:**
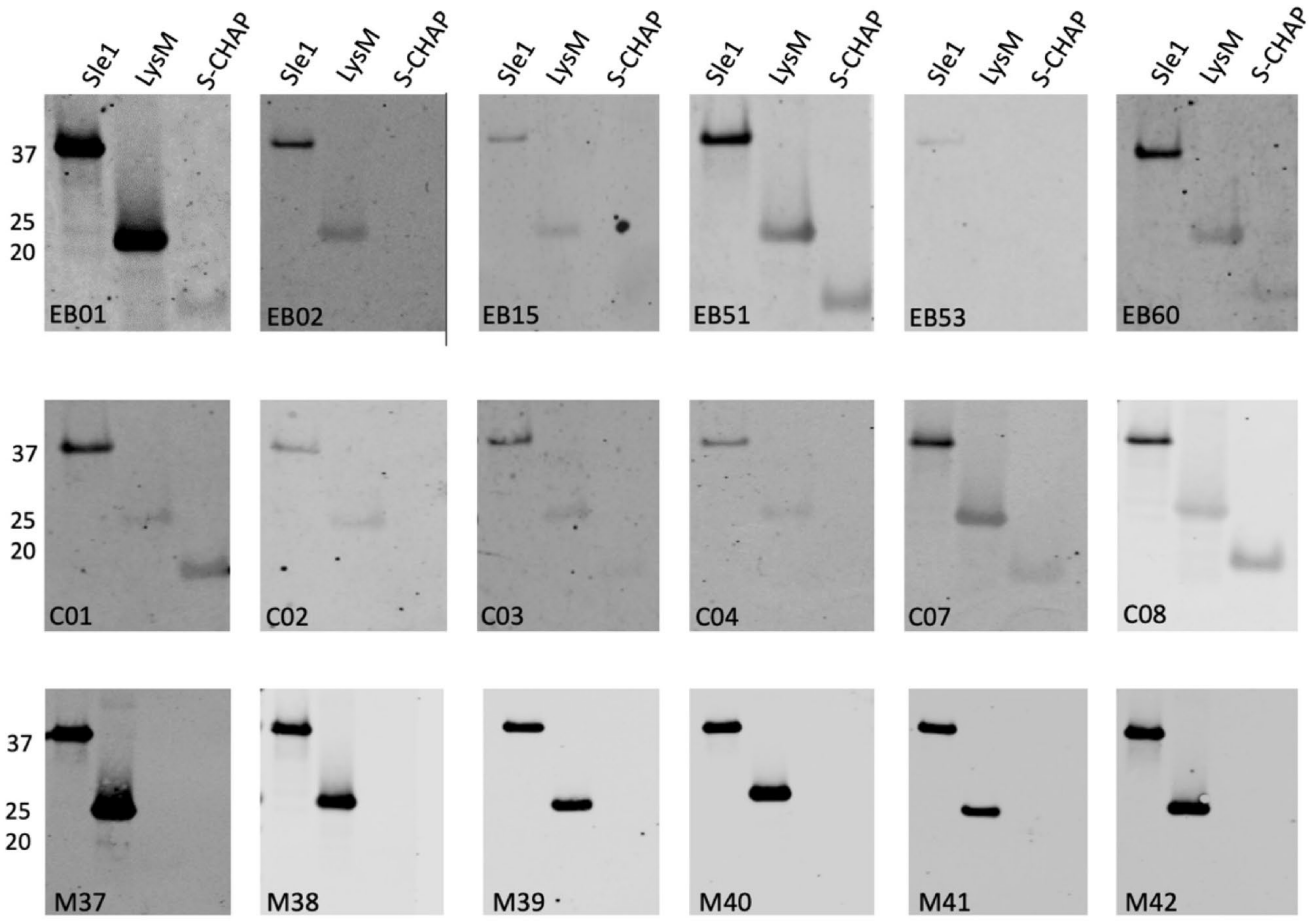
Specific binding of IgGs from EB patients, healthy volunteers and immunized mice to Sle1 and its LysM and S-CHAP domains. The purified full-size Sle1 protein and its LysM and S-CHAP domains were separated by LSD-PAGE and analyzed by Western blotting using sera from EB patients (top panels), healthy volunteers (middle panels) and mice immunized with Sle1 (bottom panels). The respective sera used for Western blotting are indicated in each panel. The molecular weights of Sle1 and its domains are indicated on the left (in kDa). For original Western blots see Supplemental Fig. 2D. The Western blotting experiments were replicated twice.

**Figure 7:**
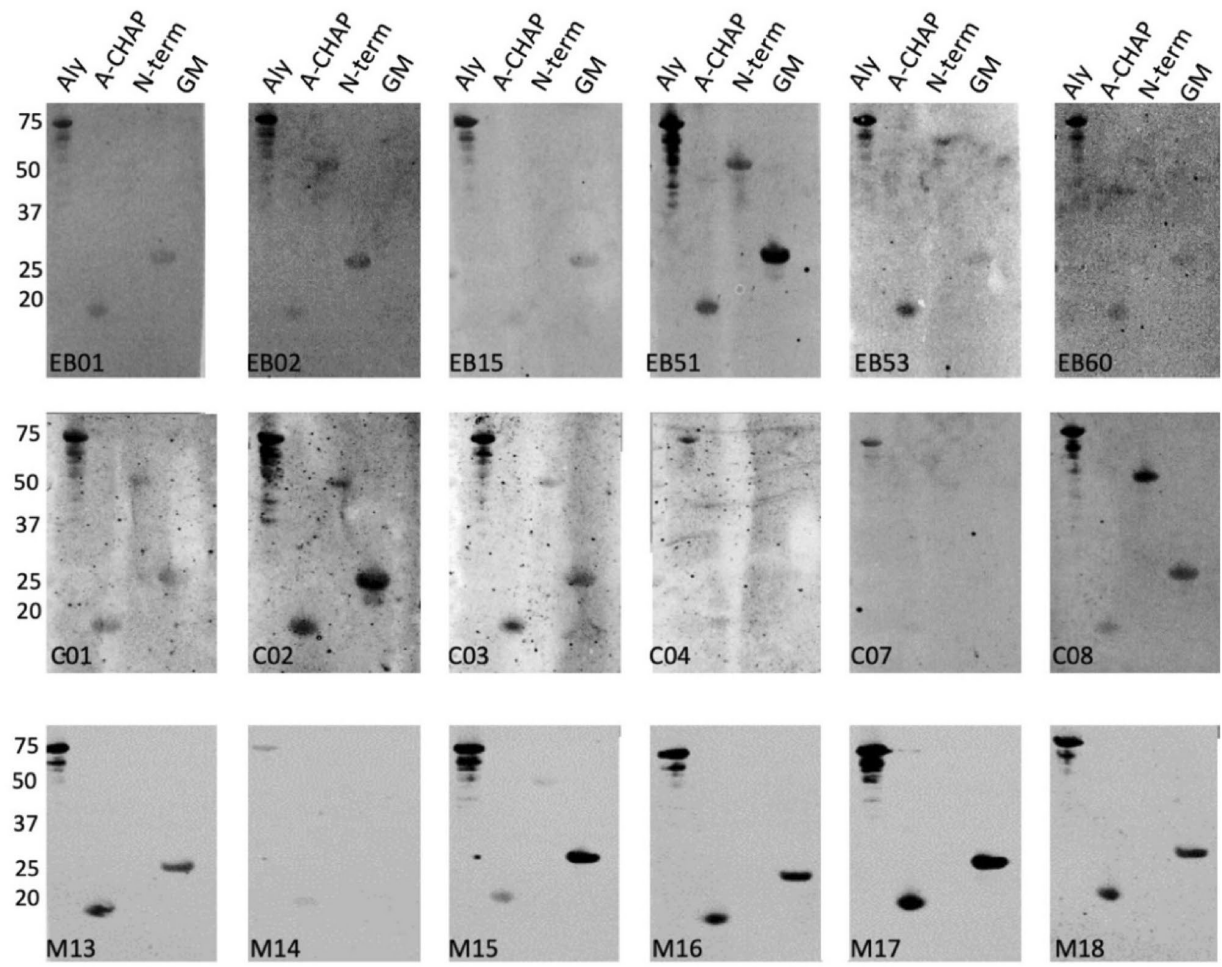
Specific binding of IgGs from EB patients, healthy volunteers and immunized mice to Aly and its A-CHAP, N-terminal and GM domains. The purified full-size Aly protein and its A-CHAP, N-terminal and GM domains were separated by LSD-PAGE and analyzed by Western blotting using sera from EB patients (top panels), healthy volunteers (middle panels) and mice immunized with Aly (bottom panels). The respective sera used for Western blotting are indicated in each panel. The molecular weights of Aly and its domains are indicated on the left (in kDa). For original Western blots see Supplemental Fig. 2E. The Western blotting experiments were replicated twice.

**Figure 8:**
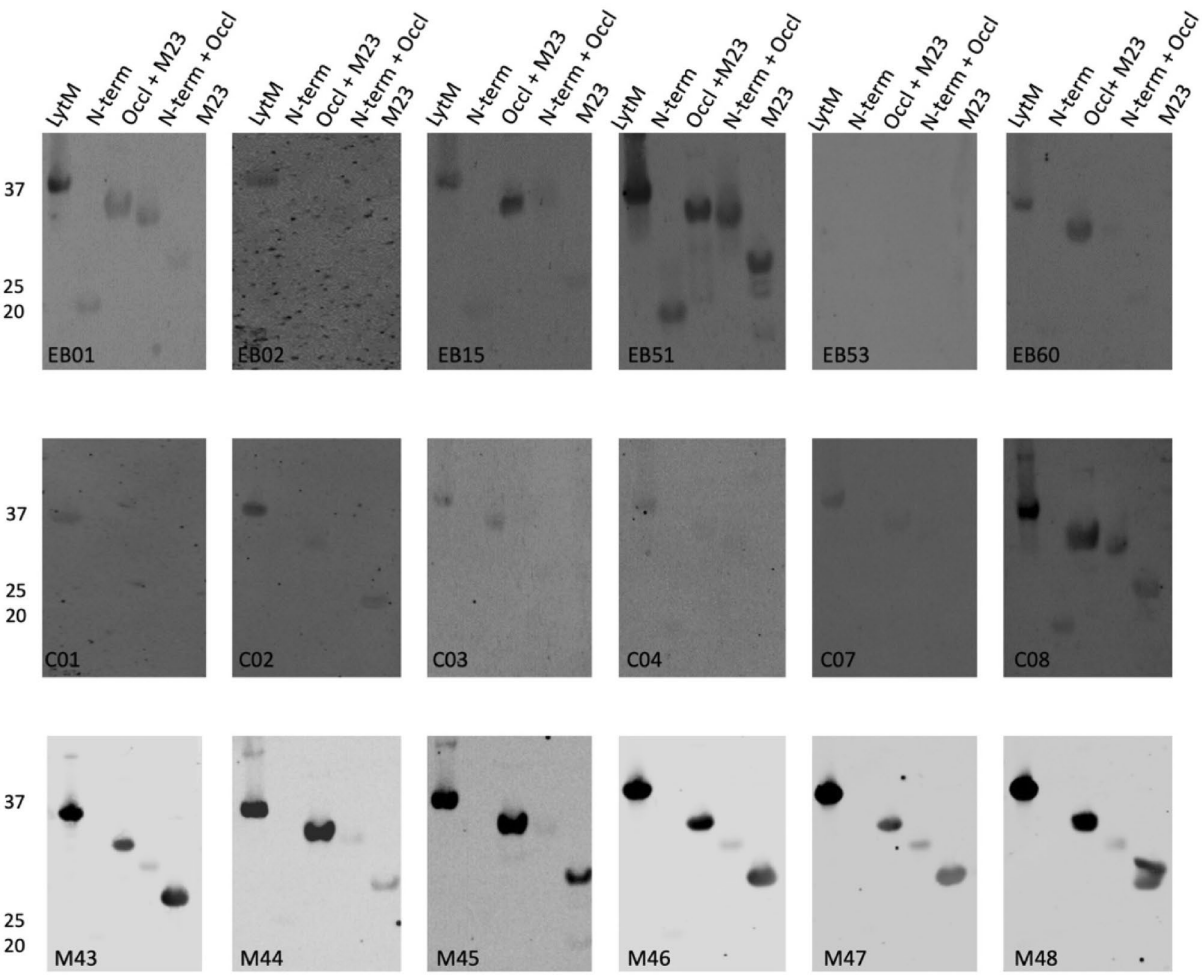
Specific binding of IgGs from EB patients, healthy volunteers and immunized mice to LytM and its N-terminal, occluding and M23 domains. The purified full-size LytM protein, and its N-terminal, occluding *plus* M23, N-terminal *plus* occluding, and M23 domains were separated by LSD-PAGE and analyzed by Western blotting using sera from EB patients (top panels), healthy volunteers (middle panels) and mice immunized with LytM (bottom panels). The respective sera used for Western blotting are indicated in each panel. The molecular weights of LytM and its domains are indicated on the left (in kDa). For original Western blots see Supplemental Fig. 2F. The Western blotting experiments were replicated twice.

To investigate possible variations in the IgG responses against Sle1, Aly or LytM in humans and mice, a Western blotting approach was applied, which included both the purified full-size proteins and their different domains. Subsequently, immunodetection was performed with sera from six EB patients, six healthy volunteers, and six immunized mice. As is evident from Figs. 6, 7 and 8, the EB patient sera contained generally higher Sle1-, Aly- or LytM-specific IgG levels than the sera from healthy volunteers. Furthermore, while the signals obtained for the different domains were more or less equal when Western blotting was performed with the His_6_-specific antibody (Fig. 2), the signals obtained with the different human sera varied substantially for the full-size proteins and their separate domains (Figs. 6, 7 and 8).

Particularly high variations were observed for the specific recognition of domains of Sle1, Aly or LytM by human IgGs (Figs. 6, 7 and 8). For instance, EB patient 51 showed high levels of IgGs specific for the Sle1, Aly and LytM proteins and their separated domains. In contrast, EB patient 53 displayed only a high level of IgGs specific for Aly and its domains, whereas IgGs specific for Sle1 or LytM were barely detectable upon Western blotting. Such individual differences were also detected for healthy volunteers. For instance, serum from the healthy volunteer C02 contained a low level of IgG specific for Sle1 and its domains, while high levels of IgGs specific for LytM and Aly were detectable (Figs. 6, 7 and 8).

Much less variation in the IgG responses was observed in the sera of the different inbred Balb/cBYJ mice immunized with Sle1, Aly or LytM (Figs. 6, 7 and 8). Only one mouse showed a lower, IgG responses to Aly (Fig. 7). However, a striking observation was that certain domains were not or barely recognized by the murine IgGs. In particular, none of the mouse sera contained IgGs binding to S-CHAP, whereas this domain of Sle1 was recognized by human IgGs (Fig. 6). Likewise, the N-terminal domain of Aly was barely recognized by the murine IgGs, whereas several human sera contained IgGs specific for this domain (Fig. 7). Also, the N-terminal domain of LytM was barely recognized by the murine IgGs and only a weak band was detectable when the N-terminal domain was tested in conjunction with the occluding domain (Fig. 8). Conversely, some of the human sera contained IgGs that did bind the N-terminal domain of LytM. Together, these observations show that both human and murine individuals displayed different IgG responses to different domains within Sle1, Aly and LytM. Furthermore, there were major differences in the IgG responses detected upon the vaccination of mice with the purified antigens, and the human IgG responses to Sle1, Aly or LytM detected as a consequence of natural exposure to these antigens due to *S. aureus* colonization of chronic wounds in the case of EB patients, or due to carriage or incidental contacts in the case of healthy volunteers.

**Figure 9:**
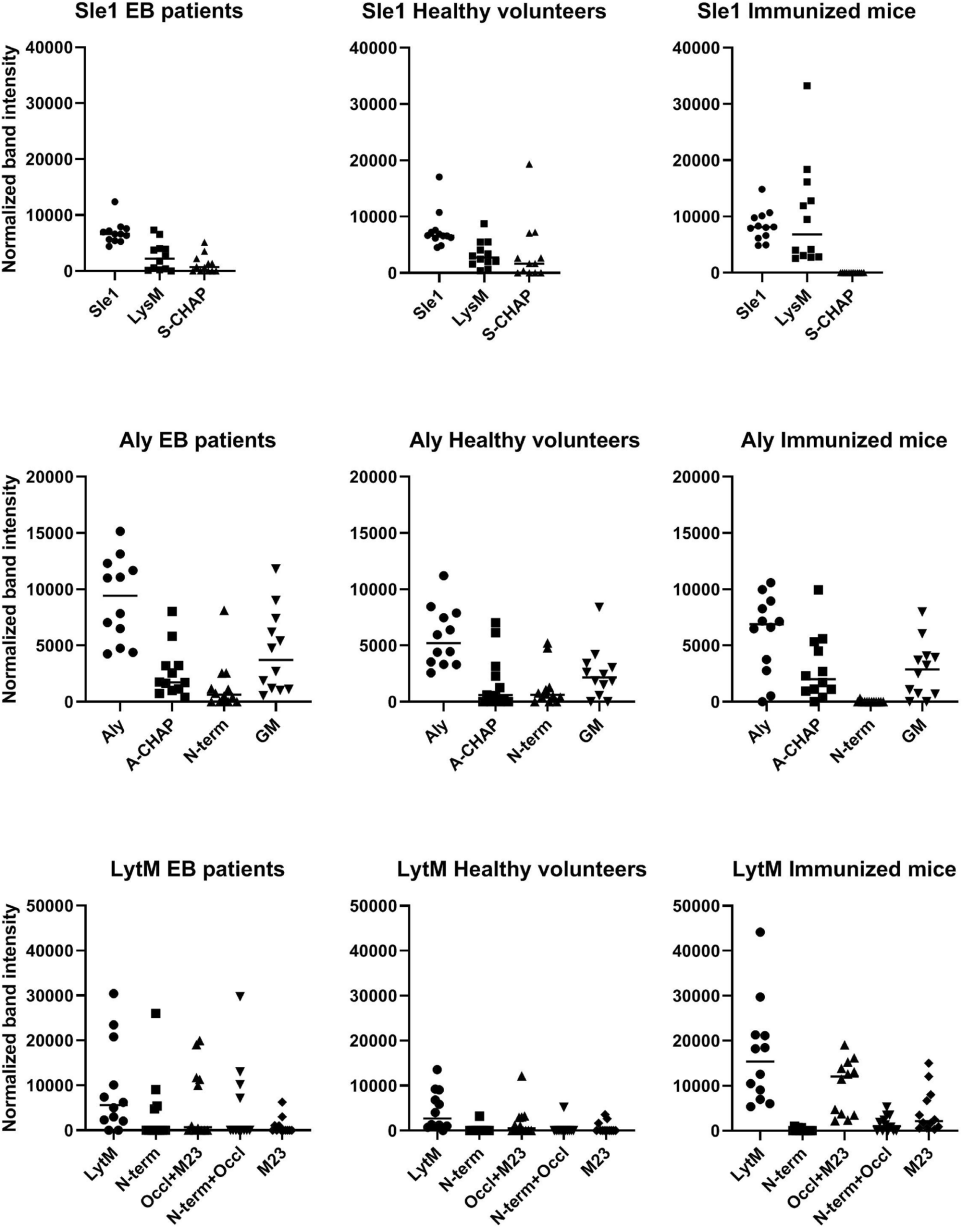
Quantification of IgG binding to Sle1, Aly and LytM full-size proteins and their domains. To compare IgG binding to the different purified proteins and their domains by IgGs from EB patients, healthy volunteers and immunized mice, the respective band intensities in Figs. 6, 7, 8 were determined with ImageJ, and normalized to the band intensities of the purified proteins and domains as determined by ImageJ analysis of the SimplyBlue Safe Stained gel presented in Fig. 2.

To be able to compare the different levels of IgG binding to the intact Sle1, Aly and LytM proteins and their different domains by IgGs from sera of EB patients, healthy volunteers and immunized mice, we analyzed the respective band intensities in the Western blots shown in Figs. 6, 7 and 8 and related them to the respective band intensities of the SimplyBlue Safe Stained gels in Fig. 2. Although the results presented in Fig. 9 show substantial variations in the IgG binding per protein and domain due to the inclusion of results obtained with sera from different individuals, this analysis highlights the differential binding observed for IgGs from humans and immunized mice.

## Discussion

In the present study we investigated the immunogenicity of the three cell wall hydrolases Sle1, Aly and LytM, using sera from healthy human volunteers, patients with EB, and mice that had been immunized with the respective proteins. In addition, we analyzed whether immunization with these cell wall hydrolases would protect mice against dying from *S. aureus* bacteremia. Interestingly, while all three cell wall hydrolases elicited significant IgG responses in the immunized mice, no protection against *S. aureus* bacteremia was observed, indicating that none of the antigen-specific IgGs was protective. This raises the question, whether human IgGs recognizing the respective cell wall hydrolases and their different domains may contribute to protection against *S. aureus* infection.

To obtain the materials needed for assessing the immunogenicity of Sle1, Aly and LytM, these three proteins and their domains were expressed in *L. lactis*, using a previously described expression system^31,36^. All proteins and their domains were well expressed and could be readily purified using a C-terminal His_6_-tag. The only exception was the occluding domain of LytM, which was not expressed for unknown reasons. However, this domain could be purified in conjunction with the N-terminal or the M23 domains of LytM. Importantly, as shown by zymography, the full-size Sle1 and its S-CHAP domain showed catalytic activity. These domains thus adapt the native, catalytically active conformation upon renaturation after SDS-PAGE, which implies that they represent *bona fide* antigens. In the case of Aly and LytM, we observed no activity for the full-size proteins, but the GM and A-CHAP domains of Aly and the M23 domain of LytM did show activity. This argues that also these domains assumed the correct conformation upon renaturation. Notably, at least in the case of LytM, the inactivity of the full-size domain can be explained based on presence of the occluding domain, which was previously shown to inhibit catalytic activity^35^. The fact that this inhibition by the occluding domain was also observed for LytM and the respective domains produced in *L. lactis* may be regarded as an (indirect) argument for correct refolding upon renaturation. At present, we do not know why the full-size Aly protein produced in *L. lactis* was not active. It is, however, conceivable that some parts of the full-size protein inhibit Aly’s enzymatic activity, similar to inhibition of LytM activity by its occluding domain. Altogether, we conclude from these analyses, that the quality of the three cell wall hydrolases and their domains produced in *L. lactis* was sufficient to use them in the present immunization and immunogenicity studies.

Immunization of mice with the purified full-size Sle1, Aly or LytM did not result in a protective immune response in the respective mice. Nonetheless, IgGs against all three proteins were detectable upon immunization. In terms of specific IgG titers, Sle1 was found to be most immunogenic and Aly the least. Further inspection of the IgG binding to particular domains of the respective proteins revealed interesting differences. For Sle1, only IgGs directed at the LysM domain were detected, for Aly only IgGs against the A-CHAP and the GM domains, and for LytM only IgGs against the M23 domain and the occluding plus M23 domain. However, the fact that the mice were not protected against death due to *S. aureus* bacteremia implies that none of the murine IgGs directed against the different investigated cell wall hydrolase domains was protective. An alternative explanation for the lack of protection could be that the Sle1, Aly and LytM proteins were not expressed by the *S. aureus* bacteria under the conditions in our murine infection model. However, we consider this possibility unlikely, because a previously published transcriptome analysis of *S. aureus* in a mouse infection model showed that Sle1, Aly and LytM are also expressed in vivo in mice^37^. Moreover, upon testing of sera of both healthy human volunteers and EB patients, we observed the presence of IgGs that recognize Sle1, Aly or LytM, which demonstrates that these three different antigens are expressed in vivo in humans. Whether the human IgGs against Sle1, Aly or LytM and their domains are also not protective is presently uncertain but, judged by our murine immunization and infection experiments, this seems unlikely. Possibly, this question could be addressed with monoclonal antibodies against the different domains of Sle1, Aly or LytM, but these are currently not available. More interesting for future immunization studies are, therefore, the human IgG responses against other domains of Sle1, Aly or LytM that did not bind murine IgGs.

The S-CHAP domain of Sle1 was specifically recognized by IgGs from healthy human volunteers and EB patients, but not by IgGs from immunized mice. Interestingly, sera from some of the healthy volunteers contained higher levels of IgG specific for the S-CHAP domain than for the LysM domain, whereas in EB patient sera the opposite trend was observed. In the case of Aly, the N-terminal domain was specifically recognized by human IgGs, but not by the IgGs from mice immunized with Aly. Lastly, also in LytM, the N-terminal domain was specifically recognized by human IgGs, although both in healthy volunteers and EB patients the respective IgG levels were relatively low. It should be noted that, at present, we do not know whether the IgGs binding to the S-CHAP domain of Sle1, and the N-terminal domains of Aly and LytM, are protective. However, it is conceivable that some of them, possibly in combination with IgGs binding other staphylococcal antigens contribute to the protection of humans against *S. aureus* infection. This would especially apply to patients with EB, whose chronic wounds are heavily colonized by *S. aureus* over extended periods of time, and who appear relatively well protected against invasive staphylococcal diseases^7^.

**Figure 10:**
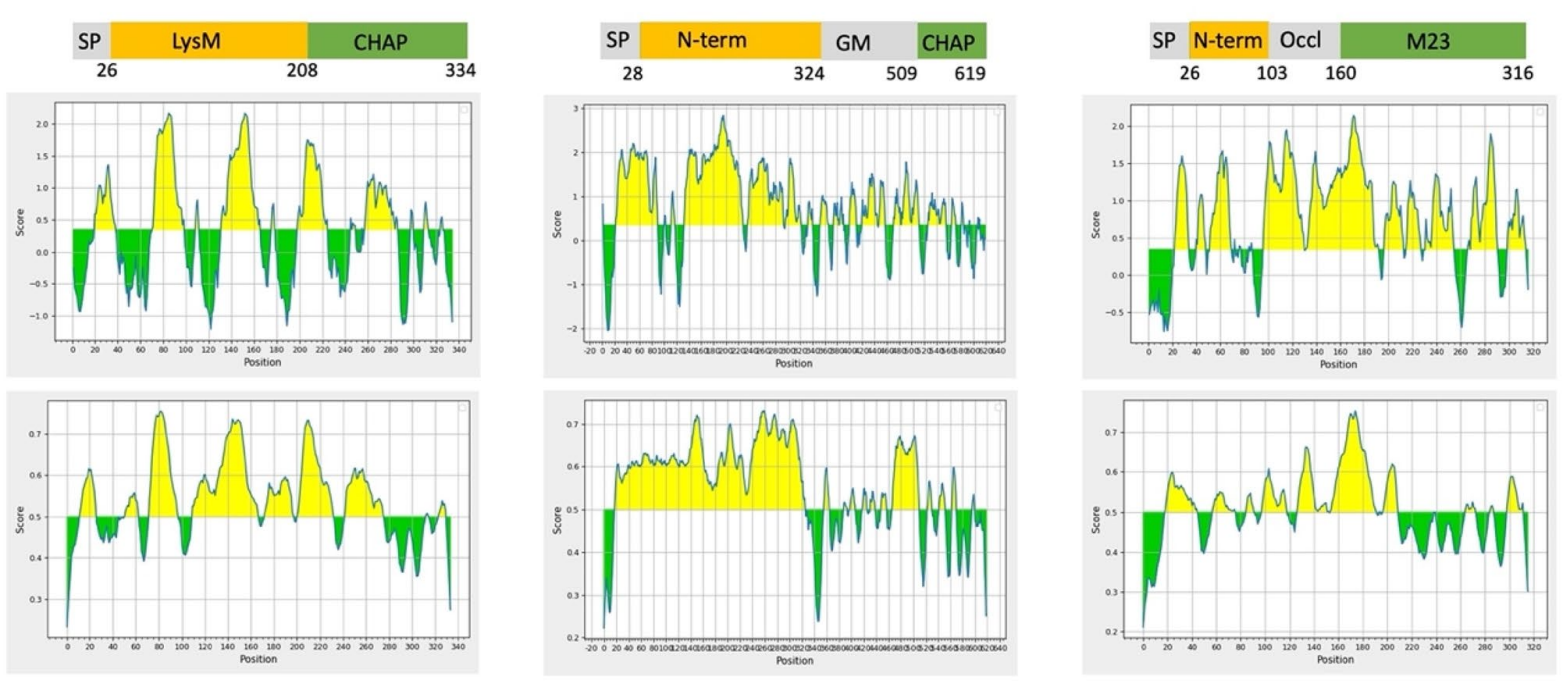
Prediction of immunogenic linear and conformational epitopes in Sle1, Aly and LytM. Linear and conformational epitopes in Sle1, Aly and LytM were predicted using the Bepipred 1.0 and 2.0 tools using the default threshold of 0.5.

An intriguing question is why humans and mice raised IgGs against different domains of Sle1, Aly and LytM. Looking at the presence of potential immunogenic epitopes predicted based on the amino acid sequence of the three proteins, it appears that all three proteins present potential linear or conformational epitopes over their entire length (Fig. 10). However, there are apparent differences in immunogenicity, as highlighted by the N-terminal domain of Aly which stands out for the high number of potential epitopes. On the other hand, the N-terminal domain of Aly was not recognized by IgGs from vaccinated mice, whereas the GM and A-CHAP domains of Aly elicited strong immune responses despite containing relatively few epitopes predicted with high confidence. This suggests that, rather than the presence of potential epitopes, the way in which the antigen is presented to the host immune system is a decisive factor for the actual immune response. Clearly, mice were vaccinated with the purified Aly protein, whereas Aly was presented to the human immune system through carriage of *S. aureus* in the case of healthy volunteers or colonization in the case of EB patients. Importantly, we have previously shown that Aly has a dual localization in *S. aureus*, being both exposed on the outer surface of the cell wall and secreted into the growth medium^38^. Possibly, presentation of Aly on the bacterial surface to the immune system elicits a better IgG response towards the N-terminal domain of this protein than when this protein is presented in a purified soluble form. Also for Sle1 a dual localization in the bacterial cell wall and extracellular milieu has been reported^25,39^. This may be a possible reason why the S-CHAP domain elicited an IgG response in humans upon presentation by *S. aureus* carriage or colonization, but not in mice upon immunization with the purified Sle1. Intriguingly, the way in which the antigen is presented to the immune system is not the only factor that determines whether epitopes elicit an IgG response to particular domains of an antigen, as is illustrated by LytM. Recently, we have shown that LytM is exclusively detectable in the growth medium of the *S. aureus* strains USA300 and Newman. This implies that *S. aureus* presents LytM to the human immune system in a soluble state, as is the case upon immunization of mice with the purified antigen. In turn, this could mean that there are molecular differences between the LytM secreted by *S. aureus* and the LytM recombinantly produced in *L. lactis*. In this respect, it should be noted that LytM was purified with a His_6_-tag for the immunization experiments, although this did not lead to His_6_-specific IgGs, which would have been detected by our Western blotting analyses. Alternatively, there could be species-specific differences in the antigen recognition by the human and murine immune systems. In this context, it is noteworthy that the A-CHAP domain of Aly elicited IgG responses both in mice and humans, whereas the homologous S-CHAP domain of Sle1 was apparently ignored by the murine immune system (Supplemental Fig. 1).

## Conclusion

Our present research shows that Sle1, Aly and LytM are highly immunogenic in humans and mice, but that the IgG-specific responses differ in the two species. Although the outcome of the murine immunization experiments was negative with respect to protection against mortality due to *S. aureus* bacteremia, these experiments provided valuable insights into the potentially most relevant domains of Sle1, Aly and LytM for the future development of protective vaccines. In particular, the S-CHAP domain of Sle1 and the N-terminal domains of Aly and LytM are interesting in this respect, although the latter domain may turn out less relevant as LytM is predominantly secreted by *S. aureu*s^38^. Importantly, the present study focuses attention on the potential use of particular domains for vaccination rather than full-size proteins. This would address potentially protective immune responses towards the most relevant domains of particular staphylococcal antigens. This view is supported by recent studies on another *S. aureus* antigen, namely IsaA, where it was observed that prophylactic administration of monoclonal IgGs specific for an N-terminal protein domain increased the time-to-death upon *S. aureus* isolate P-induced bacteremia^23,24^. In contrast, antibodies binding to other parts of IsaA were not protective. Similar results were obtained in a study by Nair et al., where the amidase domain of the major cell wall hydrolase Atl of *S. aureus* was shown to elicit a protective immune response in mice^30^. Another potential advantage of using antigen domains for vaccination rather than full-size proteins is that it may help to prevent potential unwanted immune responses against human proteins. For instance, the LysM domain as present in Sle1 is conserved both in proteins from prokaryotes and eukaryotes, including human proteins^40^. Altogether, our present studies, as well as previously published studies, focus attention on the problem that many IgG responses against *S. aureus* are not protective against this important pathogen. Such non-protective IgGs may in fact help the pathogen to evade destruction by professional phagocytes. Therefore, we urgently need to obtain a better understanding of the particular epitopes within potentially suitable antigens for vaccination that could elicit protective responses against *S. aureus*. In this respect it should be noted that also non-protective IgG responses in immunized mice are informative, as they allow the elimination of epitopes that are less likely to be successful candidates for the development of an effective anti-*S. aureus* vaccine.

## Methods

### Bacterial strains and plasmids

Strains and plasmids used in this study are listed in Table 1. *S. aureus* was grown overnight at 37 °C with continuous shaking (250 rpm) in Tryptone Soy Broth (TSB, Oxoid Limited, Hampshire, United Kingdom). *Lactococcus lactis* was grown at 30 °C in M17 broth (Oxoid Limited), or on 1% agar plates supplemented with 0.5% glucose (w/v) and chloramphenicol (5 µg/ml) for plasmid selection.

**Table 1:** Strains and plasmid used in this study.

Strain or plasmid	Characteristics	Reference
**Strains**		
*L. lactis* PA1001	Nisin induced expressing, Δ*acmA,* Δ*htrA*	^43^
*S. aureus* isolate P	Community-acquired MSSA patient isolate	^25^
*S. aureus* RN4220	Derivative of *S. aureus* NCTC8325-4	^44^
*S. aureus* N315	Hospital-acquired MRSA isolate	^45^
**Plasmids**		
pNZ::LIC::*sle1*	Fusion of pRE-USPnlic with pERL carrying *sle1* (SAOUHSC_00427, AA 26–334)	^31^
pNG400::*aly*::*his6*	pNG400 encoding Aly with a C-terminal his_6_-tag	^36^
pNG400::*lytM*::*his6*	pNG400 encoding LytM with a C-terminal his_6_-tag	^36^
pNG4210	Cm^R^, pNG400 encoding P_nisA_, SS_usp45,_ *Bam*HI-*Not*I, C-terminal his_6_-tag	^42,46^
pNG4210-*sle1*	pNG4210 encoding Sle1 with a C-terminal his_6_-tag	^47^
pNG4210-*sle1:LysM*	pNG4210 encoding LysM (AA 26–207) with a C-terminal his_6_-tag	This study
pNG4210-*sle1:CHAP*	pNG4210 encoding the S-CHAP domain of Sle1 (AA 208–334) with a C-terminal his_6_-tag	This study
pNG4210-*aly*	pNG4210 encoding Aly with a C-terminal his_6_-tag	This study
pNG4210-*aly:N-term*	pNG4210 encoding the N-terminal domain of Aly (AA 28–323) with a C-terminal his_6_-tag	This study
pNG4210-*aly:GM*	pNG4210 encoding the Glucosamidase domain of Aly (AA 324–508) with a C-terminal his_6_-tag	This study
pNG4210-*aly:CHAP*	pNG4210 encoding the A-CHAP domain of Aly (AA 509–619) with a C-terminal his_6_-tag	This study
pNG4210-*lytm*	pNG4210 encoding LytM with a C-terminal his_6_-tag	^47^
pNG4210-*lytm:N-term*	pNG4210 encoding the N-terminal domain of LytM (AA 26–103) with a C-terminal his_6_-tag	This study
pNG4210-*lytm:Occl* + *M23*	pNG4210 encoding the occluding *plus* M23 domain of LytM (AA 104–316) with a C-terminal his_6_-tag	This study
pNG4210-*lytm:N-term* + *Occl*	pNG4210 encoding the N-terminal *plus* occluding domain of LytM (AA 26–160) with a C-terminal his_6_-tag	This study
pNG4210- *lytm:M23*	pNG4210 encoding the M23 domain of LytM (AA 161–316) with a C-terminal his_6_-tag	This study

*Cm*^*R*^ chloramphenicol resistance gene, *P*_*nisA*_ nisin-inducible promoter, *His*_*6*_ hexa-histidine-tag, *SS*_*usp45*_ signal sequence of *usp45*, *AA* amino acid residues.

### Construction of plasmids for protein expression

Plasmid pNG4210 was used for the expression of different protein domains. Importantly, this plasmid carries a nisin-inducible promoter for protein expression, and the sequence encoding a C-terminal Histidine-tag (His_6_) to label the expressed protein. Primers used for cloning were obtained from Eurogentec (Maastricht, The Netherlands) (Table 2). The oligonucleotide pairs SL-F/SL-R and SCF/SCR were, respectively, used to amplify the sequences encoding the LysM- and S-CHAP domains of Sle1 (Fig. 1). The AC-F/ACH-R, AC-F/AC-R, AG-F/AG-R and ACH-F/ACH-R primer pairs were, respectively, used for amplification of the sequences encoding Aly without its natural signal peptide, the N-terminal domain, the GM domain and the A-CHAP domain. The LN-F/LN-R, LO-F/LC-R, LN-F/LO-R and LC-F/LC-R primer pairs were used for amplification of the sequences that encode the N-terminal, occluding *plus* peptidase M23 domain, the N-terminal *plus* occluding domain, and the peptidase M23 domain of LytM, respectively. Chromosomal DNA of *S. aureus* N315 was used as the template for all PCR reactions. This DNA was isolated using the Genelute bacterial genomic DNA kit (Sigma-Aldrich, Zwijndrecht, the Netherlands) following the manufacturer’s instructions. PCR reactions were performed with the *Pwo* DNA polymerase (Roche Diagnostics, Woerden, the Netherlands) using a Bio-Rad C1000 thermal cycler (Bio-Rad Laboratories, Richmond, CA). The PCR products were purified using a High Pure PCR purification kit (Analytic Jena, Jena, Germany). For cloning, the PCR-amplified fragments and the plasmid pNG4210 were cut with the restriction enzymes *Bam*HI and *Not*I (New England Biolabs, Ipswich, UK)*.* Ligation with T4 DNA ligase (New England Biolabs) was performed following the manufacturer’s protocol. The ligation products were purified using the DNA Clean & Concentrator Kits (zymo research, CA, USA), and introduced into competent *L. lactis* PA1001 cells using a Gene pulser (Bio-Rad Laboratories) as described previously^41^. Plasmid DNA from chloramphenicol resistant *L. lactis* transformants was isolated, using the innulPREP plasmid Mini kit (Analytic Jena, Jena, Germany), and the isolated plasmids were sequenced by Eurofins genomics (Ebersberg, Germany).

**Table 2:** Primers used for the construction of expression plasmids.

Primer	5′-3′ nucleotide sequence	R.E
SL-F	ATATGGATCCGCTACAACTCACACAGTAAAAC	*Bam*HI
SL-R	ATATGCGGCCGCGTTCGTAGATGCATTACCAG	*Not*I
SC-F	ATATGGATCCTCAGGATCTGCAACAACGAC	*Bam*HI
SC-R	ATATGCGGCCGCGTGAATATATCTATAATTATTTACTTGGT	*Not*I
LN-F	ATATGGATCCGCAGAA ACGACAAACACCC	*Bam*HI
LN-R	ATATGCGGCCGCTCCATTGGCATTTGCATTTTTTGG	*Not*I
LC-F	ATA TGGATCCGGAAAGGTCAATTATCCTAATGGC	*Bam*HI
LC-R	ATATGCGGCCGCTCTACTTTGCAAGTATGACGTTGGG	*Not*I
LO-F	ATATGGATCCAGCGGCCAA ACATATGTGAATCC	*Bam*HI
LO-R	ATATGCGGCCGCATTACCATCATGGCTGTTATACGC	*Not*I
AC-F	ATATGGATCCGATACACCTCAAAAAGATACTACA	*Bam*HI
AC-R	ATATGCGGCCGCAAATTGACGTGTATCTTTTGAGTC	*Not*I
AG-F	ATATGGATCCTCTAACAATGATGATAGCGGAC	*Bam*HI
AG-R	ATATGCGGCCGCATCCTTGATAGAACGTTCATATTTATC	*Not*I
ACH-F	ATATGGATCCTATGATGATTCATCAGATG	*Bam*HI
ACH-R	ATATGCGGCCGCTTTACCTGTAATATATGATAATTC	*Not*I

Restriction endonuclease (R.E.) cleavage sites are underlined.

*-F* forward primer, *-R* reverse primer.

### Overexpression, detection and isolation of proteins

For expression of full-size proteins or protein domains, *L. lactis* strains containing the respective pNG4210-based vectors, or the previously constructed plasmids pNZ::LIC::*sle*1, pNG400::*aly*::his6 or pNG400::*lytM*::his6, were grown in M17 medium. Protein expression was induced with nisin (final concentration 3 ng/ml; Sigma-Aldrich, St. Luis, MO) during the exponential growth phase when the cultures had reached an optical density at 600 nm (OD_600_) of around 0.5. Overnight growth was continued at 30 °C after which the growth medium containing the overexpressed proteins was separated from the cells by centrifugation (14,000 rpm, 4 min). Alternatively, the cells were incubated with 6 M urea for 10 min to release overexpressed full-size proteins or their domains as previously described^27^. For detection of the secreted His_6_-tagged proteins, the proteins in the collected growth medium fractions were precipitated with 10% TCA. The precipitated proteins were resuspended in lithium dodecyl sulfate (LDS) gel-loading buffer (Life Technologies, Grand Island, NY, USA) and heated for 10 min at 96 °C prior to LDS-polyacrylamide gel electrophoresis (PAGE, Life Technologies). The cell pellet obtained after centrifugation was disrupted with glass beads (0.1 μm; Biospec Products, Bartlesville, USA) in LDS gel-loading buffer using a Precellys 24 homogenizer (Bertin Technologies, Saint Quentin en Yvelines Cedex, France). Subsequently, cell debris and glass beads were pelleted by centrifugation, and proteins in the supernatant fraction were analyzed by LDS-PAGE. Lastly, proteins separated by LDS-PAGE were visualized using Simply Blue Safe Staining (Life Technologies) or Western blotting on Protran nitrocellulose membranes (Whatman, Germany). For detection of His_6_-tagged recombinant proteins or protein domains, the membranes were incubated with mouse anti-His_6_ primary antibodies (Life Technologies) at a 1:5000 dilution and, subsequently, with goat anti-mouse IRDye800CW fluorescent secondary antibodies (Life Technologies) at a 1:10,000 dilution. The His_6_-tagged proteins or domains were purified using Ni–NTA agarose beads (Promega Corporation, Madison) and quantified using the Pierce BCA Protein Assay Kit (Thermo Fisher Scientific, Waltham, USA) as previously described^31^.

### Peptidoglycan hydrolase activity assay

Zymographic analyses were performed to detect the peptidoglycan hydrolase activity of Sle1, Aly or LytM. To this end, sodium dodecyl sulphate (SDS) polyacrylamide gels (12.5%) were used, which contained 0.15% (w/v) autoclaved, lyophilized *S. aureus* RN4220 cells or *Micrococcus lysodeikticus* ATCC 4698 cells (Sigma-Aldrich, St. Louis, MO), as described previously^37^. Per sample, a total of 10 µg protein was loaded on the gel. After electrophoresis, the gels were washed with demineralized water and incubated for 18 to 36 h in renaturation buffer (25 mM Tris–HCl containing 1% Triton X-100, pH 7.2) at 37 °C with shaking. Gels were subsequently stained with 1% methylene blue in 0.01% KOH, and destained in demineralized water. Cell wall hydrolytic activities of the separated proteins or protein domains were then detectable as cleared bands in the gel. Gel images were recorded using a Xerox Phaser 3635MFP.

### Immunization of mice and detection of immune responses

Pathogen-free female BALB/cBYJ mice were obtained from Charles River (Saint-Germain-sur-l’Arbresle, France). Mice were selected as described previously^42^. For immunization, purified Sle1, Aly or LytM were emulsified 1:1 with TiterMax Gold adjuvant (Sigma-Aldrich). Immunizations were performed subcutaneously in the flank with 100 μL emulsified antigen (25 μg) on days -28, -21, and -14. Control mice received 100 μL phosphate-buffered saline (PBS) emulsified with adjuvant. Blood was withdrawn from the tail artery at day -1. IgG levels in the collected sera were examined by enzyme-linked immunosorbent assays (ELISA) using ELISA plates (Greiner Bio-One B.V, Alphen aan den Rijn, the Netherlands). The respective coating, blocking, hybridization and detection procedures were performed as described previously^37^. Bound IgGs were detected using GaM/IgG-horse radish peroxidase (HRP; SouthernBiotech) at a 1:5000 dilution in PBS-Tween, and the peroxidase reaction was visualized using o-phenylene-diamine (Sigma-Aldrich). Immunized mice (n = 6 immunized mice, n = 11 placebo-immunized mice) were challenged on day 0 by intravenous inoculation of 100 μL of *S. aureus* isolate P (3 × 10^5 ^CFU) as described previously^42^. During 14 days post infection, the discomfort and animal survival rates were monitored. To assess discomfort, clinical signs of illness in each mouse were evaluated at least twice daily as described before^42^. Accordingly, the mice were housed in individually ventilated cages with 3–4 mice per cage. The mice were given food and water ad libitum, and they were maintained in a 12:12-h light–dark cycle. Furthermore, the mice were randomly allocated to either the vaccine or the placebo groups. The mice were scored − 1 directly after bacterial inoculation, and mice with bad fur were scored − 2. Mice with bad fur and a hunched back were scored − 3. Mice with bad fur, a hunched back and showing signs of instability were scored − 4. Mice showing the latter severe signs of illness were euthanized by CO_2_ exposure.

### Detection of human and mouse IgG-binding to protein domains

To determine which domains of the expressed *S. aureus* proteins were preferentially recognized by specific IgGs from EB patients, healthy human volunteers, or immunized mice, Western blotting experiments were performed. After separation of the purified His_6_-tagged proteins by LDS-PAGE (250 ng protein/well) and subsequent transfer to nitrocellulose membranes as described above, the membranes were blocked with PBS containing 5% skim milk. To detect IgGs binding to the respective proteins or domains, sera from EB patients, healthy volunteers and immunized mice were used at a 1:2,500 dilution in PBS-Tween (20%) with 2% skim milk. Upon incubation for 1 h with the different sera and four washes with PBS-Tween, bound IgGs were detected by incubating the membranes for 30 min with goat anti-human IgG IRDye800CW or goat anti-mouse IgG IRDye800CW fluorescent secondary antibodies (LI-COR Bioscience, Lincoln, NE, USA). Subsequently, antibody binding to the membranes was inspected using an Odyssey Infrared Imaging System (LI-COR Bioscience), which allows the quantification and comparison of band intensities with ImageJ.

### Bioinformatics and data analysis

Isoelectric points (pI) and molecular weights (kDa) of proteins were predicted using the Expasy compute pI/Mw tool (web.expasy.org/compute_pi/). The statistical significance of differences in IgG titers as determined by ELISA was assessed by one-way ANOVA, further using the Bonferroni correction to determine the significance between the group. Differences in animal survival rates were analyzed using log rank tests. P-values < 0.05 were considered to indicate statistically significant differences between groups. All statistical analyses were performed using SPSS (17.0 for Windows). The BepiPred prediction server (http://tools.iedb.org/bcell/result/) was utilized to predict linear and conformational B-cell epitopes.

### Ethical approval

Sera from EB patients were collected with approval of the Medical Ethics Committee of the University Medical Center Groningen (approval no. Nl2747104209), and sera from healthy human volunteers were collected with approval of the Independent Ethics Committee of the Foundation ‘Evaluation of Ethics in Biomedical Research’ (Assen, the Netherlands). All patients and healthy volunteers provided written informed consent in this study^7^. The study was performed with adherence to the Helsinki Guidelines and local regulations. All animal experiments were performed in accordance with the rules laid down in the Dutch Animal Experimentation Act, the EU animal Directive 2010/63/EU, and the ARRIVE guidelines. The Institutional Animal Care and Use Committee of the Erasmus University Medical Center Rotterdam approved the present protocols (permit number EMC 2694).

## Supplementary Information

Supplementary Figures.
