# Supplementary material for: Differential binding of human and murine IgGs to catalytic and cell wall binding domains of Staphylococcus aureus peptidoglycan hydrolases
Source: Sci Rep. 2021 Jul 5;11:13865. doi: 10.1038/s41598-021-93359-6 (PMC8257689; doi:10.1038/s41598-021-93359-6)
Supplement: Supplementary file 1 — Supplementary Figures. [file 41598_2021_93359_MOESM1_ESM.pdf]

Supplemental Figures

Differential Binding of Human and Murine IgGs to Catalytic and Cell Wall Binding Domains of *Staphylococcus aureus* Peptidoglycan Hydrolases

Min Wang[1], Sanne van den Berg[2], Yaremit Mora Hernández[1], Aafke Hinke Visser[1], Elias Vera Murguia[1], Dennis G. A. M. Koedijk[1], Channah Bellink[1], Hilde Bruggen[1], Irma A. J. M. Bakker-Woudenberg[2], Jan Maarten van Dijl[1*], and Girbe Buist[1]

[1]Department of Medical Microbiology, University of Groningen, University Medical Center Groningen, Hanzeplein 1, P.O. box 30001, 9700 RB Groningen, the Netherlands

[2]Department of Medical Microbiology and Infectious Diseases, Erasmus University Medical Center, Rotterdam, The Netherlands.

[*]To whom correspondence should be addressed: Department of Medical Microbiology, University of Groningen, University Medical Center Groningen, Hanzeplein 1, P.O. box 30001, HPC EB80, 9700 RB Groningen, the Netherlands, Tel. +31-50-3615187, Fax. +31-50-3619105, e-mail: j.m.van.dijl01@umcg.nl

Supplemental Figure 1

A

```
S-CHAP  230  YTWGQCTYHVFNRRAEIGKGISTYWWNANNWDNAAAADGYTIDNRPTVGSIAQTDVG---  286
             Y  GQCT++V+NR  + G  IS    +A+NW+N A    Y + + P   +    + G
A-CHAP  508  YPHGQCTWYVYNRMKQFGTSISGDLGDAHNWNNRAQYRDYQVSHTPKRHAAVVFEAGQFG  567

S-CHAP  287  ---YYGHVMFVERVNNDGSILVSEMNYSAAPGILTYRTVPAYQVNNYRYI  333
                +YGHV FVE+VN+DGSI++SE N     GI+++RT+ A       YI
A-CHAP  568  ADQHYGHVAFVEKVNSDGSIVISESNVKGL-GIISHRTINAAAAEELSYI  616
```

B

```
LytM    184  GHAKDASWLTSRKQLQPYGQYHGG---GAHYGVDYAMPENSPVYSLTDGTVVQAGWSNYG  240
             GH+K    +   +  + +G+Y      G HYG+D+A+P+ +P+ + T+G V +  ++N
LytU    47   GHSKWEDFFRGSRITETFGKYQHSPFDGKHYGIDFALPKGTPIKAPTNGKVTRI-FNNEL  105

LytM    241  GGNQVTIKEANSNNYQWYMHNNRLTVSAGDKVKAGDQIAYSGSTGN-STAPHVHFQRMSG  299
             GG  + I E N   +QWY+H ++  V  GD+VKAGD IAYSG+TG  +T  H+HFQRM G
LytU    106  GGKVLQIAEDNGEYHQWYLHLDKYNVKVGDRVKAGDIIAYSGNTGKQTTGAHLHFQRMKG  165

LytM    300  GIGNQYAVDPTSYLQ  314
             G+GN YA DP  +++
LytU    166  GVGNAYAEDPKPFIE  180
```

Supplemental Figure 1. Alignment of homologous domains of Sle1 and Aly.

(A) Alignment of the S-CHAP and A-CHAP domains of Sle1 and Aly, respectively. (B) Alignment of the C-terminal catalytic M23 domains of LytM and LytU. Identical residues are indicated between the aligned sequences and conservative replacements are indicated with +.

Supplemental Figure 2

A

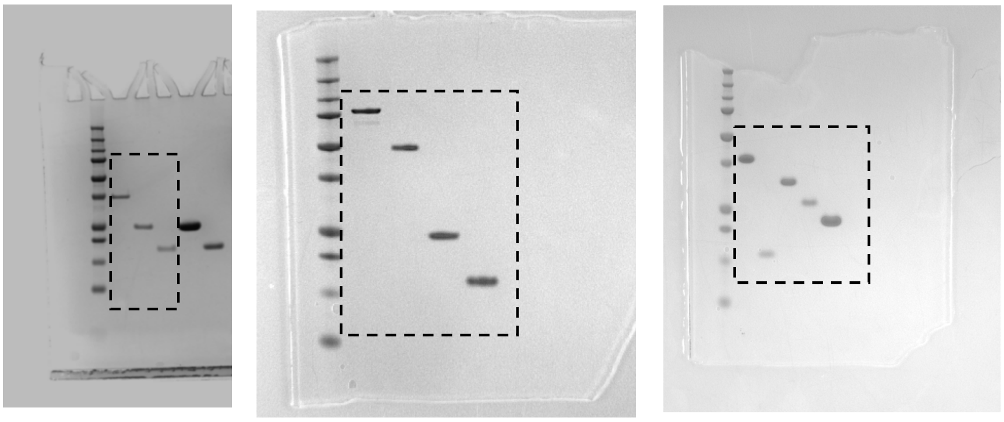

B

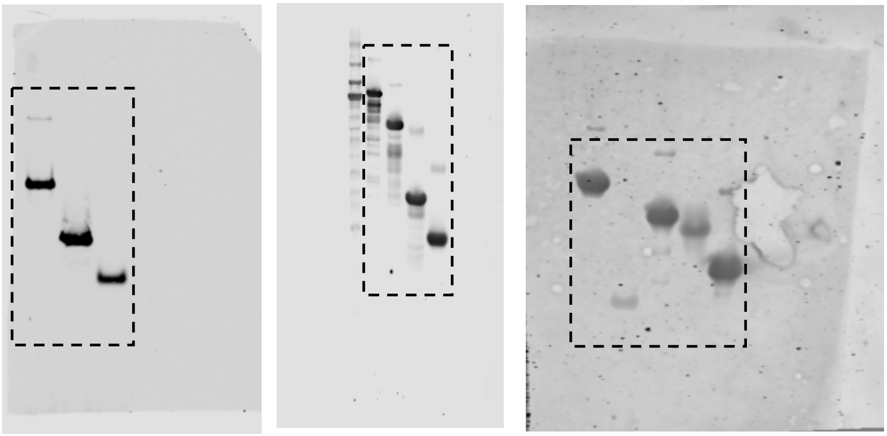

C

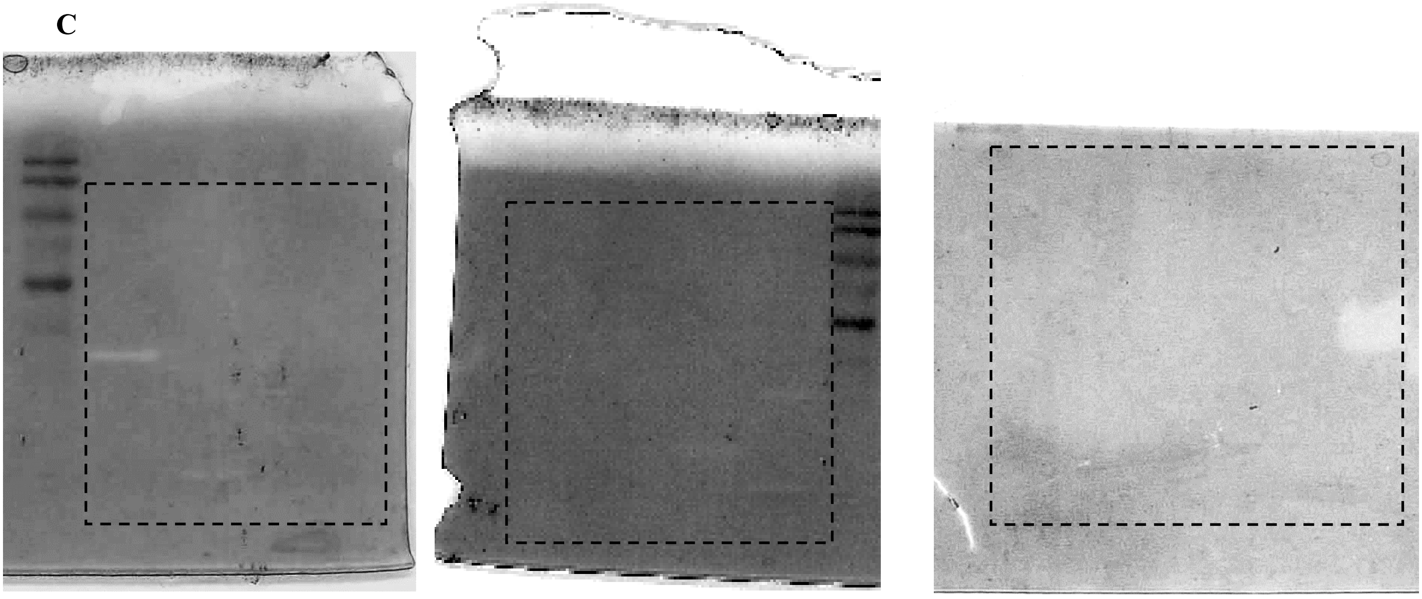

D

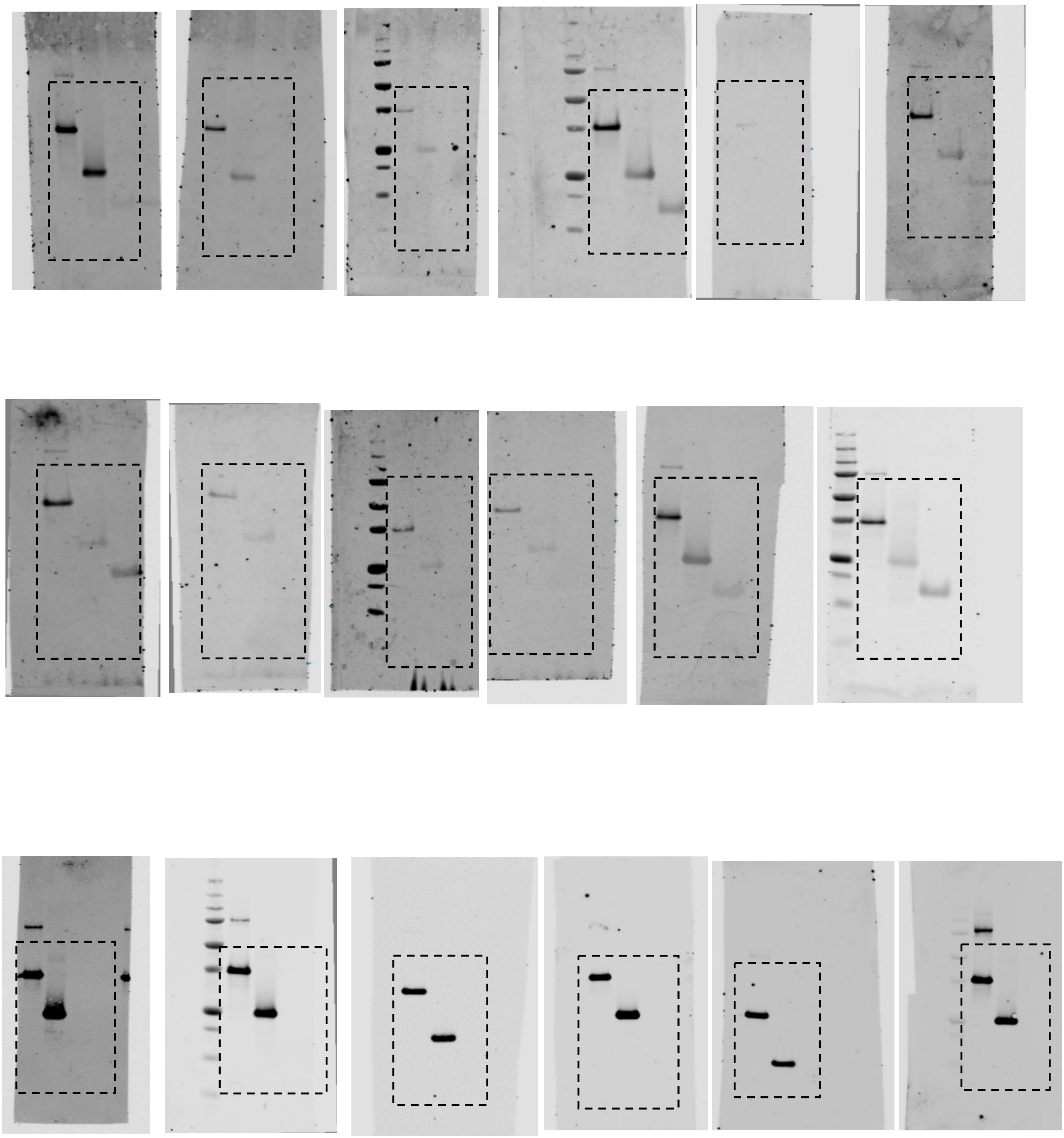

E

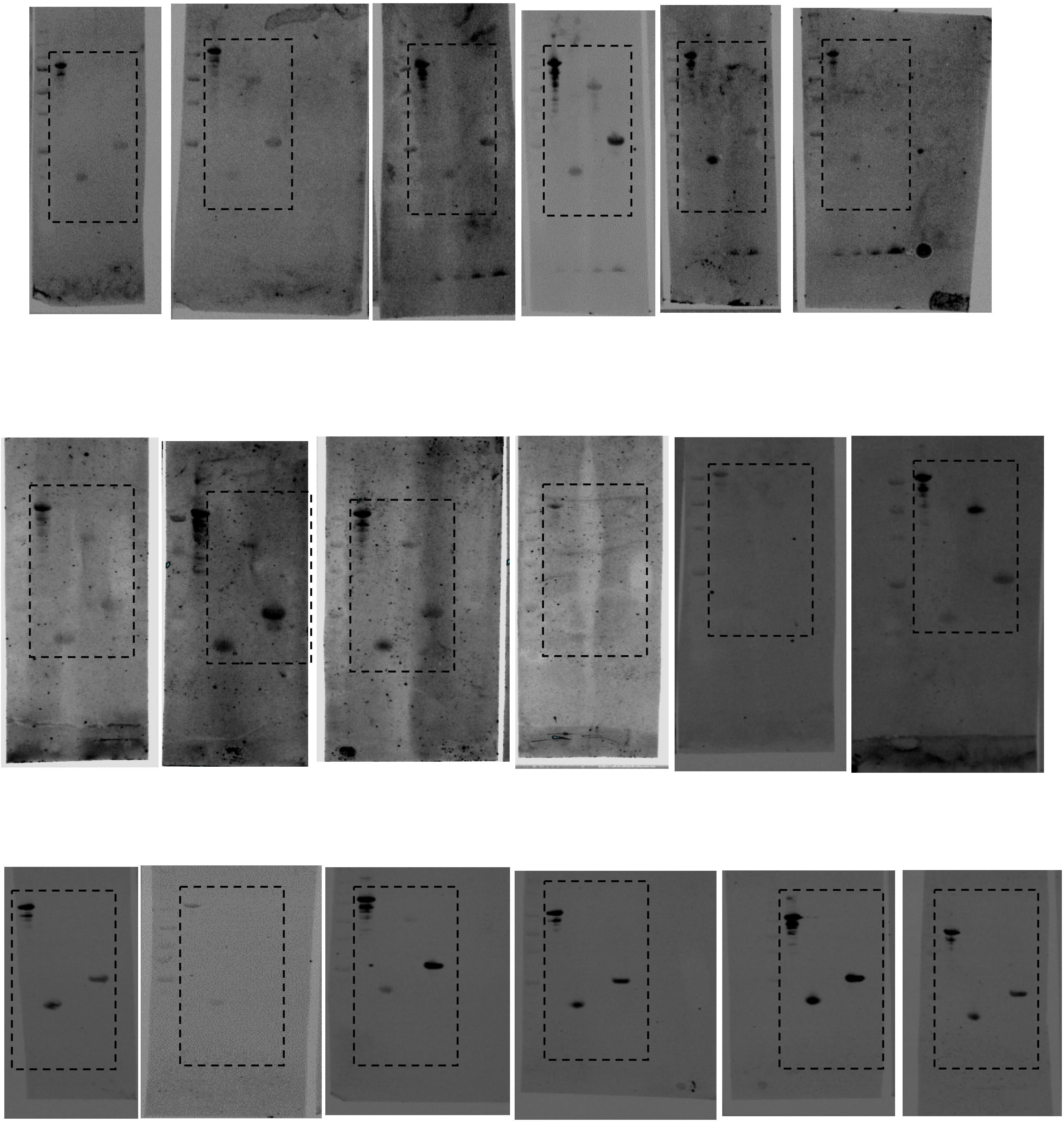

F

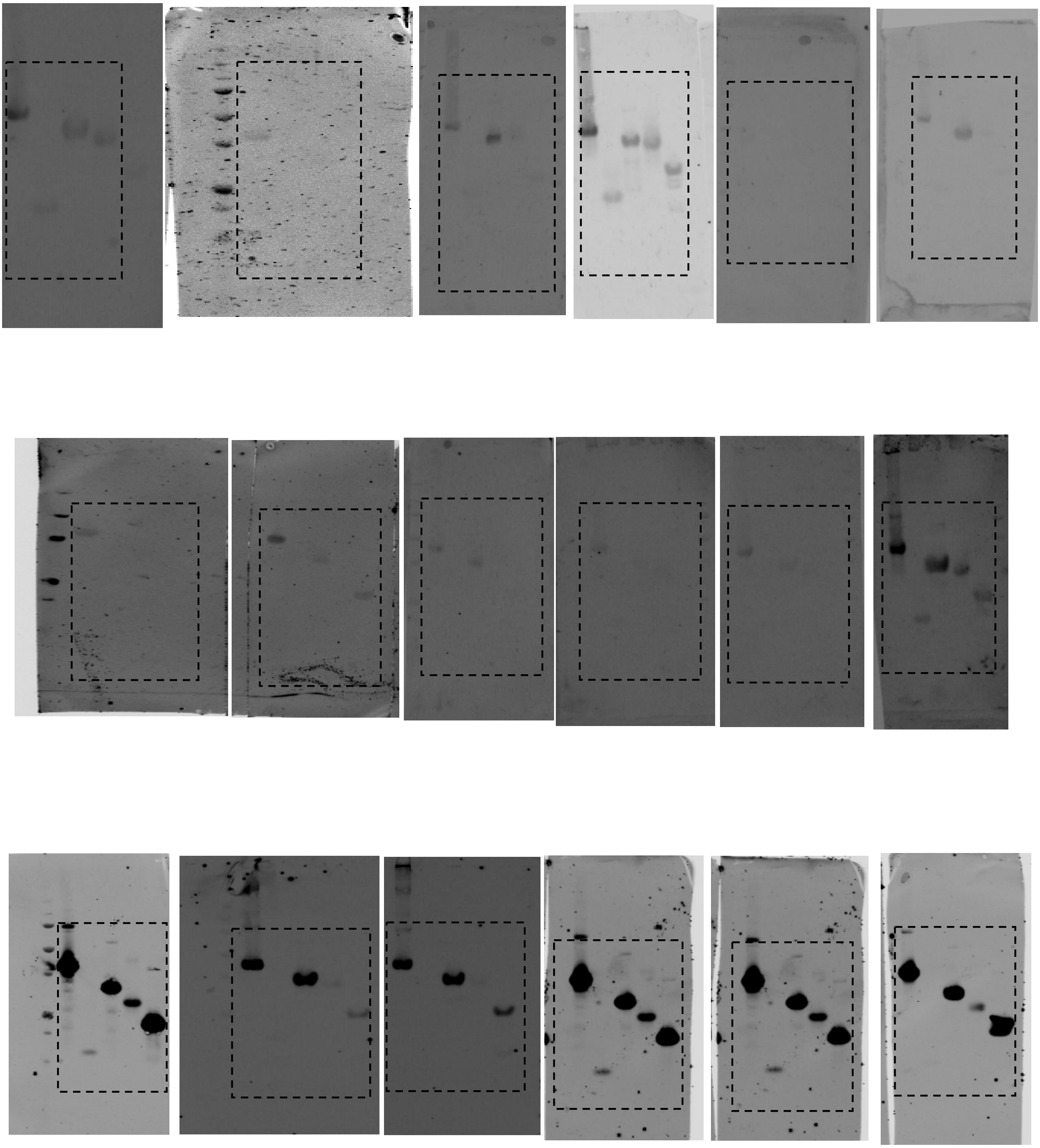

Supplemental Figure 2. Original gels and Western blots

Panel A shows the full Simply Blue-stained gels of Figure 2 (left panels).
Panel B shows the full blots used to generate the Western blots presented in Figure 2 (right panels).
Panel C shows the zymograms used to generate Figure 3.
Panel D shows the full blots used to generate the Western blots presented in Figure 6.
Panel E shows the full blots used to generate the Western blots presented in Figure 7.
Panel F shows the full blots used to generate the Western blots presented in Figure 8.
All image sections presented in the respective Figures in the main manuscript have been marked with interrupted lines.
